# Increased expression of Cks1 protein is associated with lymph node metastasis and poor prognosis in nasopharyngeal carcinoma

**DOI:** 10.1186/s13000-016-0589-9

**Published:** 2017-01-07

**Authors:** Lina Xu, Songqing Fan, Jin Zhao, Peng Zhou, Shuzhou Chu, Jiadi Luo, Qiuyuan Wen, Lingjiao Chen, Sailan Wen, Li Wang, Lei Shi

**Affiliations:** 1 Department of Pathology, The Second Xiangya Hospital of Central South University, Renmin Road 139, Changsha, Hunan 410000 China; 2 Department of Clinical Laboratory, Hunan Cancer Hospital, Changsha, Hunan China; 3 Department of Chest Surgery, The Second Xiangya Hospital of Central South University, Changsha, Hunan China

**Keywords:** Nasopharyngeal carcinoma (NPC), Cyclin-dependent protein kinase regulatory subunit 1 (CKS1), Lymph node metastasis (LNM), Prognostic factor

## Abstract

**Background:**

The Cks1 protein is an essential factor in regulating cell cycle by mediating the ubiquitination of CDK inhibitor p27^kip1^. It has been reported that aberrant expression of Cks1 and p27^kip1^ proteins was found in various tumors and related to initiation and progression of carcinomas. However, the potential roles which Cks1 and p27^KIP1^ proteins play in NPC remain unclear. This study aims to examine the expression status of Cks1 and p27^kip1^ and their possible prognostic significance in NPC.

**Methods:**

Paraffin-embedded specimens with NPC (*n* = 168) and non-tumor nasopharyngeal tissues (*n* = 49) were analyzed by IHC.

**Results:**

Expression of Cks1 increased in NPC tissues compared with non-tumor nasopharyngeal tissues (*P* < 0.05), whereas p27^kip1^ protein frequently expressed in non-tumor nasopharyngeal tissues compared with NPC tissues (*P* < 0.05). There was a significant reverse correlation between Cks1 and p27^kip1^ protein expression in NPC (*r* = −0.189, *P* < 0.05).In addition, Kaplan-Meier survival curve showed that there was a significant tendency of shorter overall survival (OS) in NPC patients with Cks1 positive expression compared to negative ones, especially in patients with lymph node metastasis (*P* < 0.001, respectively). But there was no significance between p27^kip1^ expression and survival viability of NPC patients. Multivariate Cox regression analysis further identified increased expression of Cks1 was the independent poor prognostic factor for NPC (*p* = 0.13).

**Conclusion:**

Our research found expression of Cks1 increased and was inverse to the expression of p27^KIP1^. High expression of Cks1 was significantly associated with lymph node metastasis and survival status in NPC. In addition, the abnormally high level of Cks1 protein was proved to be an independent poor prognostic factor in NPC. These results may provide novel clue for NPC therapy method.

**Electronic supplementary material:**

The online version of this article (doi:10.1186/s13000-016-0589-9) contains supplementary material, which is available to authorized users.

## Background

Nasopharyngeal carcinoma (NPC) is a common head and neck malignancy with a concentrated incidence rate in Southeast Asia compared with global distribution [1, 2]. Due to its sensibility to radiotherapy, early diagnosis and radiotherapy alone or in combination with chemotherapy have remarkably increased NPC survival rate. However, local recurrence and metastasis frequently lead to failure of clinical therapy in advanced stage patients [3]. The initiation and progression of NPC involves various factors, including Epstein-Barr virus (EBV) infection, genetic susceptibility, exposure to chemical carcinogens and mutant expression of tumor-suppressor genes, etc [4, 5]. Over the past decade, essential biomarkers involved in critical genetic events that contribute to the carcinogenesis of NPC have been increasingly found. Further investigation of novel factors associated with prognosis would provide new clue for exploring new effective therapeutic methods in NPC.

The cyclin–dependent protein kinase regulatory subunit 1 (Cks1) gene which encodes a 9KD protein Cks1 takes important roles in cell growth, proliferation and apoptosis. The protein Cks1 is an essential adaptor of the SCF-SKP2 E3 ligase ubiquitin ligase complex which appends ubiquitin to targets for degradation through the ubiquitin proteasomal system [6]. One of crucial functional roles of Cks1 is to mediate the ubiquitination of CDK inhibitor p27^Kip1^ and lead to promote cell cycle progression from G1 to S phase [7, 8]. Aberrant expression of Cks1 and p27^KIP1^ has been found in multiple human cancers and is significantly associated with tumor invasion and metastasis [9–13]. Although Cks1 expression is generally inversely related to p27^Kip1^ expression, but some studies reported different expression pattern of Cks1 and p27^Kip1^ proteins which emphasize potentially p27^Kip1^ independent mechanisms of Cks1 in cancer progression [14, 15]. The relevance between Cks1 and p27^Kip1^ protein and the clinicopathological characteristics in NPC remains unclear. In this present study, we aimed to identify the expression level of Cks1 and p27^KIP1^ in NPC and their potential relationship with clinicopathological features.

## Methods

### Tissue specimens

In this study, 168 cases of paraffin-embedded tissue from the primary NPC patients and 49 cases of nasopharyngeal mucosa tissues from patients with chronic nasopharyngitis were obtained from the Second Xiangya Hospital of Central South University (Changsha, China). The collection of specimens and study protocol were approved by the Institutional Human Experiment and Ethics Committee of the Second Xiangya Hospital of Central South University.

Complete clinical record and follow-up data from all NPC patients were collected. None of NPC patients received radiotherapy or chemotherapy prior to diagnosis. Among 168 cases of NPC patients, there were 14 cases of differentiated non-keratinizing nasopharyngeal carcinoma and 154 cases of undifferentiated non-keratinizing nasopharyngeal carcinoma. The clinical stages and treatments of all patients were as follows: 3 cases of clinical stage I, 42 cases of stage II, 77 cases of stage III and 46 cases of stage IV; 78 patients treated with radiotherapy, and 90 patients treated with combined radiotherapy and chemotherapy. Among these NPC patients in this study, total 94 patients (55.9%) were alive with a mean follow-up period of 67 months (3–120 months).

### IHC staining

Total of 168 formalin-fixed, paraffin- embedded NPC and 49 non-tumor nasopharyngeal mucosa tissues were collected for immunohistochemical analysis. Briefly, each section was deparaffinized, rehydrated, high-temperature retrieved by heating slides in citrate buffer (pH 6.0) at 100 °C for 4 min, bathed in 0.3% H_2_O_2_ in methanol for 30 min and blocked by 10% preimmune goat serum for 30 min. The slides were incubated with a 1:200 dilution of primary antibody of Cks1 (Sigma Aldrich) or a 1:200 dilution of primary antibody of p27^Kip1^ (Abcam Inc.) at 4 °C over-night, and then stained by Ready-to-use Envision Dual Link System-HRP methods (Dako Inc.) according to the instruction. Color reaction was visualized with Diaminobenzidine (DAB) solution. Counterstaining was carried out with hematoxylin. Besides the internal positive control, the positive and negative control slides were contained in each experiment.

Scoring all slides were performed as described in our previous publication [16]. All slides were scored manually by two independent individuals at 200 magnification light microscopy.

### Statistical analyses

All statistical analyses were performed with SPSS 19.0 software. The significance of Cks1 or p27^Kip1^ proteins expression in NPC and non-tumor tissues was tested by *χ*2-test. The relevance between expression of Cks1 and p27^Kip1^ proteins in NPC was analyzed by Spearman’s rank correlation coefficient. The association between expression of Cks1 and p27^Kip1^ proteins and clinicopathological features in NPC was assessed by the *χ*2-test. Survival curves were constructed by using the Kaplan–Meier method and statistical significance was assessed by the log-rank test. The prognostic significance of expression of Cks1 and p27^Kip1^ proteins was evaluated by the Cox proportional hazard regression model. *P*-value of <0.05 was considered statistically significant.

## Results

### Expression of Cks1 protein increased and p27^kip1^ protein decreased in NPC

**Fig. 1:**
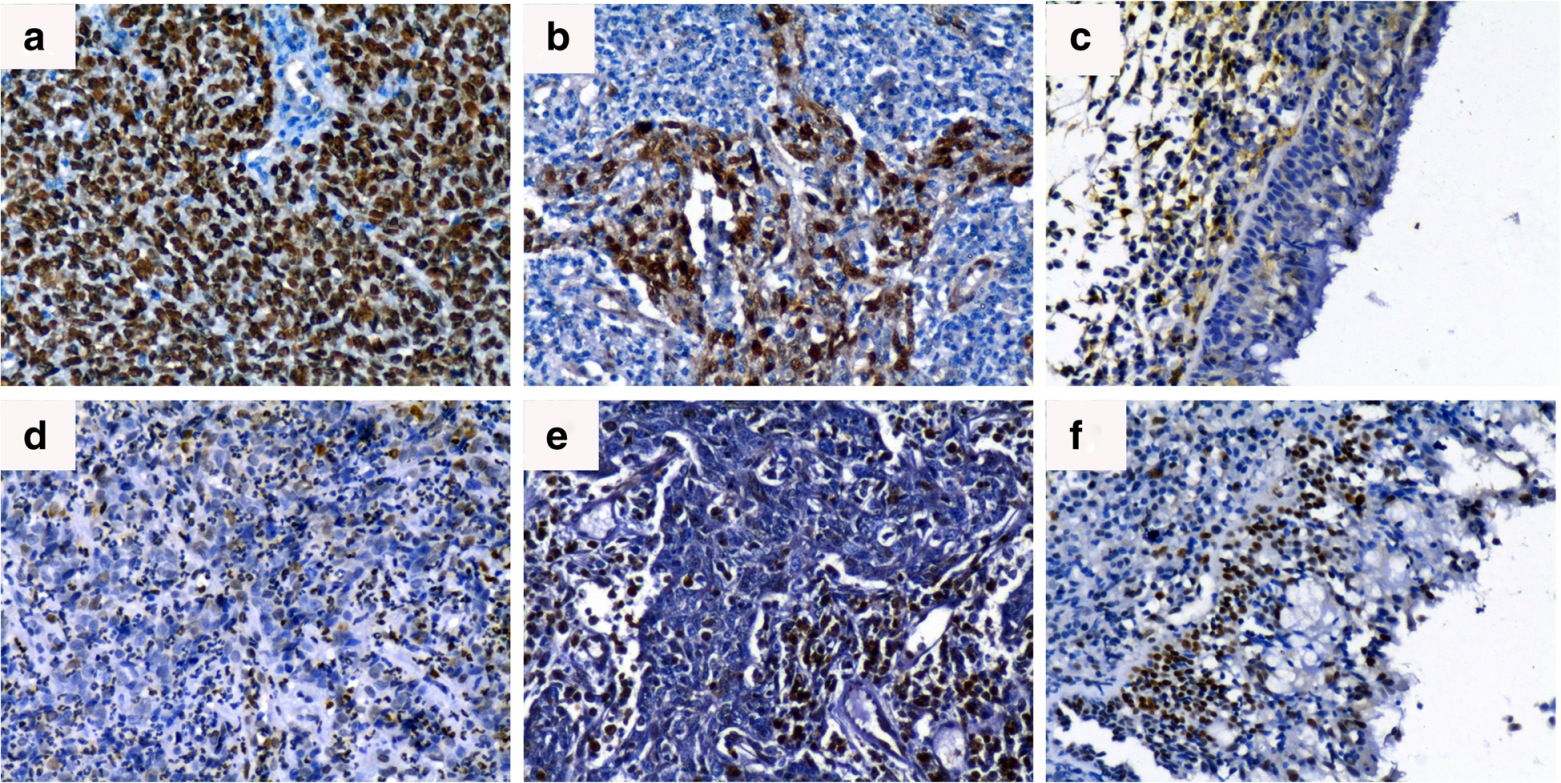
Expression of Cks1 and p27^KIP1^ in NPC and non-tumor nasopharygeal tissues by IHC. Strong staining of Cks1 protein was identified in cell nuclei of primary NPC tissues (**a**) and lymph node metastasis tissues (**b**). Negative staining of Cks1 protein was identified in the normal nasopharyngeal epithelia (**c**). Moderate staining of p27^KIP1^ was found in primary NPC tissues (**d**) and lymph node metastasis tissues (**e**). Strong staining of p27^KIP1^ was found in cell nuclei of non-tumor nasopharygeal epithelium (**f**)

To clarify the significance of Cks1 and p27^kip1^ proteins in NPC, we investigated the expression of Cks1 and p27^kip1^ in NPC and non-tumor nasopharyngeal tissues by IHC method. As shown in Fig. 1, positive staining of Cks1 protein mainly localized in the nucleus and in 73.2% of NPC tissues (Fig. 1a), especially in tumor metastasized in lymph node (Fig. 1b) and 36.7% of normal nasopharyngeal tissues (Fig. 1c). Staining of p27^kip1^ protein was not strong as cks1 protein did in tumor tissues (Fig. 1d and e), but was positively expressed in 57.1% of normal nasopharyngeal epithelial tissues (Fig. 1f). As shown in Table 1, the statistical data confirmed that Cks1 protein highly expressed in NPC tissues compared with non-tumor nasopharyngeal tissues (73% vs. 36%, *P* < 0.05), and p27^kip1^ protein frequently expressed in non- tumor nasopharyngeal tissues compared with NPC tissues (57% vs. 43%, *P* < 0.05).

**Table 1:** Expression of Cks1 and p27^KIP1^ in NPC and non-tumor nasopharygeal tissues

Variable	Cks1 Expression			p27^kip1^ Expression		
	P (%)	N (%)	*p-value	P (%)	N (%)	*p-value
NPC (*n* = 168)	123 (73.2)	45 (26.8)	.001	23 (13.7)	145 (86.3)	.001
Non-tumor Nasopharyngeal (*n* = 49)	18 (36.7)	31 (63.3)		28 (57.1)	21 (42.9)	

*Abbreviations: P* positive, *N* negative

* Note: Chi-square test, *p* <0.05

To examine the relevance between cks1 and p27^kip1^ protein in NPC, we analyzed data with Spearman’s correlation. In Table 2, the result illustrated that expression level of Cks1 and p27^kip1^ protein was significantly inverse in NPC (*r* = −0.189, *P* < 0.05).

**Table 2:** The pairwise association between expression of Cks1 and p27^kip1^ in NPC

	Cks1	p27^kip1^
Cks1 Spearman’s Correlation Coefficient Sig. (2-tailed)	1	−0.189*
p27^kip1^ Spearman’s Correlation Coefficient Sig. (2-tailed)	−0.189*	1

*Note:* Values are Spearman’s correlation coefficient

* Correlation is significant at the *p* <0.01 level (2-tailed)

These study results confirmed that expression of Cks1 increased in NPC, and was companied with a reverse expression of p27^kip1^ protein.

### Increased expression of Cks1 was associated with clinicopathological features of NPC

To explore if the mutant expression of Cks1 and p27^kip1^ was associated with clinical outcomes of NPC, we utilized univariate chi-square test to verify the relevance between Cks1 and p27^kip1^ proteins expression and NPC clinicopathological characters. The Table 3 indicated that NPC tissueswith lymph node metastasis presented higher expression of Cks1 than those without lymph node metastasis (*p* = 0.007), and NPC tissues from patients of the deceased group possessed higher expression of Cks1 than the alive group (*p* < 0.001). However, no significant correlation was found between the expression of Cks1 and other clinicopathological features, including age, gender, histological type and clinical stage (*p* > 0.05, respectively). The expression of p27^KIP1^ was not related to any of clinicopathological features. This result proved that increased expression of Cks1 protein was associated with lymph node metastasis and survival status.

**Table 3:** Association between expression of Cks1 and p27^KIP1^ and clinical pathological features of patients with NPC

Characteristics	Cks1			p27kip1		
	P (%)	N (%)	*p*-value	P (%)	N (%)	*p*-value
Age (yr)						
≤ 50 (*n* = 101)	73 (72.3)	28 (27.7)	.859	15 (14.9)	86 (85.1)	.382
> 50 (*n* = 67)	50 (74.6)	17 (25.4)		8 (11.9)	59 (88.1)	
Gender						
Female (*n* = 41)	32 (78)	9 (22)	.421	4 (9.8)	37 (90.2)	.399
Male (*n* = 127)	91 (71.7)	36 (28.3)		19 (15.0)	108 (85.0)	
Histological Type						
DNC (*n* = 14)	11 (78.6)	3 (21.4)	.636	3 (21.4)	11 (78.6)	.379
UDNC (*n* = 154)	112 (72.7)	42 (27.3)		20 (13.0)	134 (87.0)	
Clinical Stage						
Stage I-II (*n* =45)	30 (66.7)	15 (33.3)	.246	5 (11.1)	40 (88.9)	.556
Stage III-IV (*n* =123)	93 (75.6)	30 (24.4)		18 (14.6)	113 (78.6)	
LN Status						
LNM (*n* = 125)	111 (88.8)	14 (11.2)	.007*	16 (12.3)	109 (87.2)	.567
No LNM (*n* = 43)	30 (69.8)	13 (30.2)		7 (16.3)	36 (83.7)	
Survival Status						
Alive (*n* = 94)	57 (60.6)	37 (39.4)	.000*	13 (13.8)	81 (862)	.953
Dead (*n* =74)	66 (89.2)	8 (10.8)		23 (13.7)	145 (86.3)	

*Abbreviations: DNKC* differentiated non-keratinized nasopharyngeal carcinoma, *UDNC* undifferentiated non-keratinized nasopharyngeal carcinoma, *LN* lymph node, *LNM* lymph node metastasis, *P* positive, *N* negative

*Note:* chi-square test, *: *p* < 0.05

### Aberrant expression of Cks1 protein predicted poor diagnosis in NPC

**Fig. 2:**
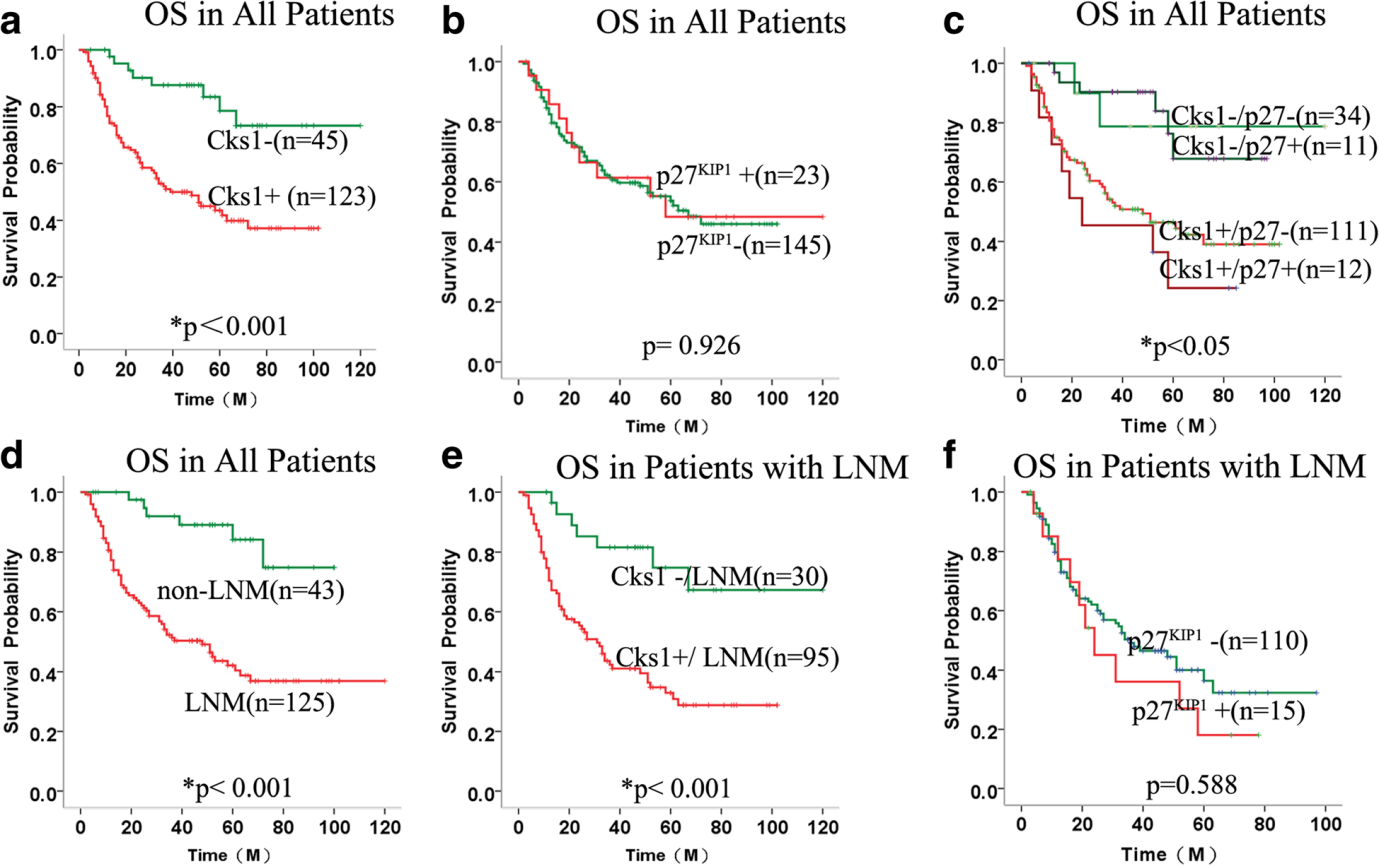
Kaplan-Meier curves for overall survival of the NPC patients. **a** Kaplan-Meier survival curves for OS in NPC patients with Cks1 positive and negative expression. **b** Kaplan-Meier survival curves for OS in NPC patients with p27^KIP1^ positive and negative expression. **c** Kaplan-Meier survival curves for OS in 4 group NPC patients: Cks1-positive & p27^KIP1^-negative, Cks1-positive & p27^KIP1^-positive, Cks1-negative & p27^KIP1^-negative and Cks1-negative &p27^KIP1^-positive. **d** Kaplan-Meier survival curves for OS in NPC patients with and without LNM. **e** Kaplan-Meier survival curves for OS in group of LNM patients with Cks1 positive and negative expression. **f** Kaplan-Meier survival curves for OS in group of LNM patients with p27^KIP1^ positive and negative expression. Statistical significance was assessed using the log-rank test

To further evaluate the effect of increased expression of Cks1or decreased expression of p27^kip1^ in the survival of NPC, the Kaplan-Meier survival curve of all 168 NPC patients was constructed by the Kaplan-Meier analysis. As shown in Fig. 2, Kaplan-Meier survival curves depicted that overall survival (OS) in NPC patients with Cks1 positive expression was shorter compared with the negative ones (*P <* 0.001, log rank =16.466) (Fig. 2a), but there was no significance between expression of p27^kip1^ and NPC patients survival rate (*p* > 0.05) (Fig. 2b). The combined analysis of Cks1 and p27^kip1^ expression revealed that the expression level of p27^KIP1^ protein did not affect the relevance between Cks1 and the survival of NPC patients (Fig. 2c). In addition, compared with those patients without LNM, patients with LNM showed a tendency of poor prognosis (*P* < 0.001, log rank = 19.556) (Fig. 2d). After patients being stratified by LNM, patients with Cks1 positive expression showed significantly shorter OS than negative group (*P* <0.001, log rank = 15.056) (Fig. 2e), while the significance of p27^KIP1^ in NPC patients survival was not found (Fig. 2f).

Furthermore, we carried out multivariate Cox proportional hazard regression analysis to estimate the prognostic value of Cks1 or p27^kip1^ protein in NPC. The clinical stage, T-stage (volume of tumor), lymph node metastasis status, histological type, treatment strategy, age and gender, as well as expression of Cks1 and p27^kip1^ proteins were included in the multivariate analysis of 168 cases of NPC. Shown in Table 4, results confirmed that increased expression of Cks1 was identified as an independent poor prognostic factor for NPC (*P* < 0.05), as did as clinical stage, treatment strategy and LNM status (*P* < 0.05, respectively). However, neither expression of p27^kip1^ protein or other clinicopathological features was detected to be the independent prognostic factors for NPC (*P* > 0.05, respectively). This result hinted that high expression of Cks1 protein might be a poor prognostic factor in NPC.

**Table 4:** Summary of multivariate analysis of Cox proportional hazard regression for overall survival in NPC

Parameter	Wald	Sig	Exp (B)	95.0% CI for Exp (B)	
				Lower	upper
Cks1	6.185	.013 *	2.652	1.230	5.721
P27^KIP1^	1.174	.279	1.486	.726	3.042
T-stage	1.271	.260	.835	.611	1.142
Clinical Stage	7.624	.006 *	1.783	1.183	2.689
LNM Status	15.04	.000 *	7.086	2.635	19.06
Histological Type	2.101	.147	.496	.192	1.280
Treatment Strategy	8.461	.004 *	3.549	1.512	8.332
Age	.000	.999	1.000	.607	1.646

*Abbreviations: LNM* lymph node metastasis, *CI* confidence interval, *P* positive, *N* negative

*Note:* multivariate analysis of Cox regression,*p <0.05

## Discussion

The alterations of expression or activity of proteins which is related to cell cycle regulation are of extensive interest, because uncontrolled proliferation is a critical character in tumor progression. Cyclin-dependent kinase (CDK) inhibitor p27^Kip1^ inhibits the activity of G1-cyclin–CDK complexes and arrests cell-cycle progression in G1 phase. P27^Kip1^ is degraded in the late G1 phase via the ubiquitin–proteasome pathway [17]. The Cks1 protein is a member of the highly conserved family of Cks/Suc1 proteins which interact with Cdks and participates in numerous cellular processes including cell proliferation, growth and survival [18, 19]. One of well established mechanisms of Cks1 modulating cell cycle is to bind with the C-terminal of Skp2 to degrade p27^Kip1^ and to promote cell cycle progression from G1 to S phase [7, 8, 18]. Recent evidence has revealed that Cks1 is over-expressed in a majority of tumors, solid tumors such as gastric carcinoma [20], oral squamous cell carcinoma [21], colorectal carcinoma [22], salivary gland tumors [9], esophagus carcinomas [10], hepatocellular carcinoma [11], breast cancer [12], non-small cell lung carcinoma [23], and hematologic tumors, like lymphoma [14], multiple myeloma [13]. In those studies, high expression of Cks1 is associated with tumor formation and aggressiveness. It is frequently observed that high Cks1 expression is correlated with high SKP2 and low p27^Kip1^ and is associated with tumor progression in some cases. Those studies coincide with our results and further indicate the important roles of Cks1 in tumor progression.

In this study, we found that the expression of Cks1 increased in NPC tissue, and high expression of Cks1 protein was correlated with LNM status and survival status in NPC patients. But, p27^Kip1^ expression was not correlated with the clinicopathological characteristics in NPC, although IHC analysis result showed that p27^Kip1^ expression was attenuated in NPC patients and statistical analysis data confirmed that the expression of p27^kip1^ was inversely related to Cks1 in NPC. The non-significance of p27^Kip1^ in prognostic evaluation of tumor has been observed in esophagus carcinomas [10], hepatocellular carcinoma [11], and non-small cell lung carcinoma [23] too. Those evidences support our results and suggest that Cks1 might influence progression of NPC through p27^KIP1^-independent ways.

In fact, some studies also indicate Cks1 is involved in regulating cell cycle transitions by other targets, not only p27^KIP1^. For instance, Cks1 promotes cell to enter from G0 to G1 by mediating the ubiquitination of CDK1 inhibitor p130 in breast cancer [15]. Besides the mechanisms of regulating cell cycle, a few studies have improved that Cks1 is required in cell transcriptional events. Cdc20 has been reported to be a transcriptional target of Cks1 [24]. Cks1 protein is primarily involved in modulating the transcriptional activation of the APC/C protein-ubiquitin ligase activator Cdc20 to promote mitosis [24, 25]. Cks1 has also been reported to transcriptionally regulate the expression of cdc2, cyclin B and cyclin A in mammalian cells [26]. Moreover, the involvement of Cks1 in MAPK [27], JAK-STAT [28] and NF- κB [29] cell signaling pathways has been reported recently. Cks1 influences cell proliferation and apoptosis through activating the phosphorylation of MEK1/2 and ERK1/2 in breast cancer, and the phosphorylation of MEK1/2, ERK1/2 and STAT3 in multiple myeloma [28]. Our research results suggested that Cks1 might promote NPC invasion and progression through multiple ways, not only by p27^Kip1^-dependent mechanism.

In recent years, due to radiotherapy and chemotherapy resistance, the effect of treatment in NPC is at a standstill. Increased expression of Cks1 promotes the radiation resistance ability of ESCC cells [30]. Cks1 over-expression leads to multidrug resistance in multiple myeloma cells in vitro by activating MAPK and STAT3 pathways [28]. But, enforced expression of Cks1 enhanced chemotherapeutic sensitivity by overriding DNA damage checkpoints in breast cancer cell in vitro and in vivo [31]. These inconsistent research results suggest that Cks1 protein might play complex roles in mechanism of tumor cell tolerance to radiotherapy and chemotherapy. Our research results uncovered that high expression of Cks1 was correlated with lymph node metastasis and survival rate in NPC, which may offer potential target for effective treatment in NPC.

## Conclusion

In summary, we first reported that the expression of Cks1 protein increased and p27^KIP1^ decreased in NPC. Over-expression of Cks1 was associated with the poor overall survival rate of NPC. Furthermore, multivariate analysis suggested that high expression of Cks1 protein might be regarded as the independent prognostic factor for poor prognosis in NPC patients. These results may provide novel clue for NPC therapy methods.

## Additional file

Additional file 1: The original data of IHC scoring. The expression of CKS1 and p27KIP1 had been tested in 168 cases of paraffin-embedded tissue from the primary NPC patients and 49 cases of non-cancerous nasopharyngeal control specimen from independent patients by IHC. The percentage of positive cells was divided into five grades (percentage scores):<10% (0), 11–25% (1), 26–50% (2), 51–75%(3), and >75% (4). The intensity of staining was divided into four grades (intensity scores): no staining (0), light brown (1), brown (2),and dark brown (3). Staining positivity was determined by the formula: overall scores = percentage score × intensity score. The total score ranged from 0 to 12, with negative staining (0–1) and positive expression (2–12). At last, 0 presented negative staining and 1 presented positive staining in the data file. (XLS 42 kb)

## Abbreviations

CKS1: Cyclin-dependent protein kinase regulatory subunit 1
DNKC: Differentiated non-keratinized nasopharyngeal carcinoma
LN: Lymph node
LNM: Lymph node metastasis
N: Negative
NPC: Nasopharyngeal carcinoma
P: Positive
UDNC: Undifferentiated non-keratinized nasopharyngeal carcinoma
